# Sex and Speciation: Drosophila Reproductive Tract Proteins— Twenty Five Years Later

**DOI:** 10.1155/2012/191495

**Published:** 2012-10-17

**Authors:** Rama Singh, Santosh Jagadeeshan

**Affiliations:** ^1^Department of Biology, McMaster University, Hamilton, ON, Canada L8S 4K1; ^2^Smithsonian Tropical Research Institute, P.O. Box 0843-03092, Balboa, Panama

## Abstract

The protein electrophoresis revolution, nearly fifty years ago, provided the first glimpse into the nature of molecular genetic variation within and between species and showed that the amount of genetic differences between newly arisen species was minimal. Twenty years later, 2D electrophoresis showed that, in contrast to general gene-enzyme variation, reproductive tract proteins were less polymorphic within species but highly diverged between species. The 2D results were interesting and revolutionary, but somewhat uninterpretable because, at the time, rapid evolution and selective sweeps were not yet part of the common vocabulary of evolutionary biologists. Since then, genomic studies of sex and reproduction-related (SRR) genes have grown rapidly into a large area of research in evolutionary biology and are shedding light on a number of phenomena. Here we review some of the major and current fields of research that have greatly contributed to our understanding of the evolutionary dynamics and importance of SRR genes and genetic systems in understanding reproductive biology and speciation.

## 1. Introduction

Science aims to provide simple and general explanations for natural phenomena, and all sciences must deal with the problem of heterogeneity. Variation and heterogeneity are the hallmarks of biological diversity and capture the attention of anyone interested in trying to unravel the mysteries of the biological world. Explaining biological diversity was indeed the problem for which Darwin provided a simple but revolutionary solution [1]. Variation and adaptation are the first two words that come to mind in relation to living organisms, and it was Darwin's genius that in using these two observations he was able to formulate the theory of natural selection to explain the diversity that we see reflected in the millions of different kinds of organisms or species on this planet. Given its spectacular success in providing a causal explanation for organismic change (evolution within lineages), it is equally remarkable that Darwin was unable to provide a causal mechanism of speciation (evolution between lineages). Such a causal theory had to wait nearly a century after the publication of Darwin's *Origin of Species* [1] and it materialized only after the evolutionary synthesis of the 1940s after population genetics had developed a theoretical framework [2, 3].

## 2. Speciation Genetics: Mendelian versus Molecular Approach

Diversity is a problem in biology in two ways: the most obvious of which is that diversity needs to be explained; the other is that it can thwart our efforts in elucidating precise and simple explanations for the complexity of biological phenomena. As a consequence, we trade precision for generality [4] and a rich theoretical base has been developed, which at first appears to ignore diversity. Population genetics is a good example as it developed a significantly elaborate theory of how genes change in evolution without prior knowledge of the material/chemical basis of the gene or of genetic variation. This is also true of speciation as well. The allopatric theory of speciation relied on geographic isolation and differentiation of populations [3] and cumulative effects of gene differences and gene incompatibility [2, 5] without specifying anything about the nature of the genes involved. It was therefore surprising that when gel electrophoresis made its debut in scientific methods, genetic differences between closely related species turned out to be minimal [6]. Much of this had to do with the fact that the genes being investigated by molecular biologists were of the general cell metabolism category (i.e., allozymes) and had no direct relation to reproductive biology—the “crime scene” of reproductive isolation. On the other hand, the Mendelian approach to studying speciation focused on the right phenotype—hybrid dysfunction— and the breakdown in the sexual machinery of hybrids. The Mendelian approach therefore eventually became more successful in speciation research by uncovering regions of chromosomes and discrete genes with large effects that played a role in causing hybrid sterility/inviability [7].

These so-called candidate “speciation genes” provided a glimpse into the nature and the variety of genes that effect postzygotic reproductive isolation, but by the virtue of deliberately being chosen as “large effect” genes (for the ease of mapping), they may or may not represent the pool of genetic variation that is the basis of speciation. It is for this reason that another, parallel approach to investigating the nature of genetic variation affecting reproductive isolation was needed. To understand the genetic basis of reproductive isolation, a more systematic methodology was needed to screen genes and genetic variation associated with the reproductive system. A systematic genomic/proteomic approach was essential because not all genes in the reproductive system would affect reproductive isolation; many are indeed essential for development and reproductive biology. We needed to find and target the genes and proteins in the reproductive system that matter—genes with minor or large effects that are most likely involved in the early stages of species isolation.

It was indeed this realization that led us to the idea of investigating genetic variation in the reproductive tracts of *Drosophila*. The idea of using 2D electrophoresis to examine reproductive proteins seemed exciting but there was some hesitation due to the technical difficulties associated with the technique. Mike Coulthart, a graduate student at that time, was up to the task as he had what was needed: technical precision, patience, and perseverance. He investigated the levels of genetic variation and genetic differentiation, respectively, within and between sibling species of the *Drosophila melanogaster* group. 

The 2D results were surprising and somewhat un-interpretable at the time. By separating over 250 protein spots from the reproductive tract and comparing them between species, Mike found little genetic variation within species but high genetic divergence between species [8–10]. Under the neutral theory we expected parity between the levels of within-species and between-species variation, which was indeed the case in the massive amount of data that had accumulated using one-dimensional gel electrophoresis [6, 11]. The 2D results were therefore interesting and revolutionary given the dominant framework of neutrality and the expected constant and slow rate of evolution. These novel data raised many interesting questions but received little attention. This was mainly because reproductive tract proteins were considered essential to the organism and therefore not expected to evolve rapidly. 

The unusual nature of these results called for more investigations and research on sex and reproduction-related (SRR) molecules began. The ensuing series of experiments involving 2D electrophoresis showed that (1) nonenzymatic, abundant proteins were generally less polymorphic than enzymes; (2) reproductive tissue proteins were more diverged between species than nonreproductive tissue proteins, such as those of the brain [12]; (3) testes and ovary proteins showed higher levels of species divergence than nonreproductive proteins [13]; (4) reproductive proteins (and reproductive morphological traits) showed more gene expression breakdown in species hybrids than nonreproductive proteins [13–16]. These data and particularly Civetta and Singh's [13, 14] research, which showed that sex and reproduction-related (SRR) genes evolve faster than nonreproductive genes, unveiled the importance of studying the evolution of SRR molecules in speciation research [14, 17–20]. 

## 3. DNA Sequence Variation and Rates of Evolution

While the average rates of evolutionary change per gene may be small, genes can evolve rapidly depending upon the environmental conditions and the selection pressure. The dynamics of selection acting on each locus will determine its rate and pattern of evolution. Some groups of genes may evolve rapidly by virtue of their functions as is the case with the immune response genes in mammals [21]. Immune response genes are an example of a coevolutionary system where evolution of immunity or resistance in hosts is countered by the evolution of virulence in pathogens and/or parasites. Immune system genes and virulence genes are locked in an antagonistic coevolutionary arms race and are expected to evolve rapidly [22]. Sexual system genes provide another example of a coupled coevolutionary system, in this case involving the interactions between males and females of the same species. 

### 3.1. Rapid Evolution of SRR Genes

Advances in molecular technology particularly DNA amplification and sequencing propelled SRR gene research and a remarkable trend of pervasive rapid SRR gene evolution emerged at several levels. Some sex determining genes, assumed to be conserved due to their important functions during early development, were shown to evolve rapidly [23]. When genes expressed in testis, ovary, and nonreproductive tissues were screened for rates of evolution it became clear that a substantial proportion of these genes evolved more rapidly than genes expressed in nonreproductive tissues [24, 25]. A divergence trend of testis > ovary > somatic genes emerged suggesting male and female SRR genes evolve under different selective pressures [26]. Microarray and computational methods using entire tissue-specific transcriptomes showed that testis that expressed SRR genes were more likely to break down in species hybrids [27, 28]. Rapid SRR gene evolution was also found in gametes. Sperm proteins were shown to evolve rapidly and divergently in invertebrates and mammals [29–32]. Proteins in the seminal fluid of *Drosophila* were also found to be evolving rapidly and were shown to confer specific physiological and behavioural modifications in the female [33–37]. These studies not only indicate that sexual reproduction provides an opportunity for exerting constant selection pressure generation after generation but also that differences in the evolutionary dynamics of male and female reproductive systems are presumably due to intersexual selection pressure arising from male-female interaction in each generation. The relationship between rapid evolution of SRR genes and reproductive isolation is attested by the fact that the known candidate “speciation genes” are either SRR genes or autosomal genes that, via incompatible interactions with genes on the X chromosome, affect hybrid dysfunction (reviewed in [38–41]). In addition, genome-wide evidence of rapid evolution of SRR genes provided a mechanistic framework to discuss the nature of genetic changes that may occur during speciation [14, 17, 42].

### 3.2. Evolution of New SRR Genes

 The discovery of *jingwei* [43] and *Sdic* [44] opened up investigations into the origin of new SRR genes marking yet another important step into understanding the evolutionary dynamics of genetic systems [45–48]. Novel genes arise through a variety of molecular mechanisms, including being derived from previously noncoding DNA [49] and may be important in functional diversification. What is extremely interesting is that the majority of novel genes or gene copies that have evolved novel functions have also evolved testis-specific expression. Interestingly, *Odysseus* (*OdsH*), a hybrid sterility gene, evolved as a duplicate of the neuron expressed *unc-4* gene and has taken up a testicular expression and role [50, 51]. Another example is *ms(3)K81,* a gene that evolved by duplication and retroposition from a previously ubiquitously expressed copy to acquire a male-germline-specific expression and function [52]. *ms(3)K81* is only found in the 9 species of the melanogaster subgroup and in its new functional role is crucial for zygote viability [52]. Accessory gland proteins (*Acps*) are a prime example of male-specific genes in *Drosophila* that have taken up a variety of reproduction-related functions and have important physiological effects in the female reproductive system [34–37, 53–56]. Retrotransposition is another important means of gene copying and shuffling that can be important in the evolution of new functions [45, 46]. Interestingly more genes moving from the X chromosome to autosomes have been retained (active) than genes moving in the opposite direction [7, 47, 57–61]. Again, testis-specific expression and rapid evolution appear to be common amongst retroposed genes. Thus for some yet unexplained reason, it appears that the testes act as cauldrons of retaining, if not manufacturing, new/refugee genes. In fact, it turns out that not only the evolution of new genes but also gene loss (loss of orthology) is also elevated in male-biased genes as compared to female-biased genes in *Drosophila* species [27]. The genetic machinery of the sexual system shows faster rates of turnover and it points to the role of sexual selection acting preferentially through the males (male-driven sexual selection) [62, 63]. The evolution of new genes and novel functions can be a potent driving force of reproductive isolation as exemplified by *Odysseus* (*OdsH*). 

## 4. Evolution of Sex-Biased Genes: Role of Sexual Selection versus Selection in relation to Sex

Sexual selection, strictly speaking, is only a small part of the total selection pressure that the organism is exposed to in relation to sex. Classical theories of sexual selection apply only to those traits and genes that are influenced, directly or indirectly, by female choice. On the contrary, selection in relation to sex, or what has been called “sexual selection in the broad sense” [64], applies to all aspects of reproductive biology—from soliciting mates, courting, and mating, to production of offspring. A large proportion of the genome is involved in the development and maintenance of reproductive systems and a significant proportion of genes (~30%) in the *Drosophila* genome shows sex-biased gene expression, most of which is reproductive tissue specific [65, 66]. This raises the possibility of an unexpected level of conflict between natural and sexual selection. Current studies of sexual selection have expanded to different aspects of reproductive biology and constitute a major area of research in evolutionary biology. A few examples are discussed below.

### 4.1. Sexual Dimorphism and Sex-Biased Gene Expression

Sexual dimorphism is common and often dramatic amongst animal taxa. Despite being “genetically” identical (with the exception of the Y-chromosome), males and females are expected to differ in genes associated with primary sexual characteristics such as ovary, testes, and copulatory organs. Traditionally thought to be associated to few genes on the sex chromosome, it turns out that the breadth and complexity of sexual interaction between the two sexes has become so elaborate that a large number of genes controlling a variety of traits have become associated in a sex-specific manner (sex-biased and sex-enriched genes) expanding the level of sexual dimorphism [66, 67]. In the *Drosophila* genome a substantial proportion of genes show sex-biased expression [65, 66]. Male-biased genes are underrepresented on the X chromosome and female-biased genes are enriched on the X chromosome. These genes are expressed in a tissue-specific manner (e.g., somatic tissues, ovary, and testis) and they even show sex-specific elevated levels of movement between sex chromosome and autosomes [59, 60, 67–72]. In the last decade, several theories including sexual antagonism, dosage compensation, meiotic sex chromosome inactivation, and meiotic drive have been proposed to explain the paucity of male-biased gene on the X chromosome and the driving force responsible for the evolution of sex chromosomes, their gene content, and expression patterns [58, 67, 72–74]. Meiotic sex chromosome inactivation (MSCI) pioneered first by Lifschytz and Lindsley [75] and then shown at a genomic scale [58, 72] appeared to be convincing in *Drosophila*; however, recent evidence shows otherwise and is currently under debate [74, 76–79]. While explaining the relocation and expression pattern of sex-biased genes will remain a prominent research area, it is noteworthy that, with respect to rates of evolution, genes with sex-biased expression and particularly male-biased sex genes show unusually high rates of evolution [67, 80, 81]. It is not surprising then that the sexes differ in their rates of evolution of sterility and inviability during speciation as pointed out by Haldane [82]. 

### 4.2. Evolution of Egg-Sperm Interaction

Sea urchins have traditionally been a model organism for development biology and reproductive biology but have recently received considerable attention from an evolutionary standpoint—particularly in the evolution of reproductive isolation to explain speciation in the sea [83, 84]. In most internally fertilizing animals, specific courtship behaviours mediate male and female interactions ensuring species-specific copulation and fertilization. In contrast, in externally fertilizing organisms with little or no such mating behaviours (e.g., sea urchins) gametes are shed into the sea where species-specific fertilization occurs. This requires the evolution of elaborate molecular mechanisms that ensure specific-specific fertilization. Several molecules on the surface of gametes that mediate various stages of sperm-egg interaction have been characterized [85–87]. Studies on two important proteins exemplify the evolutionary dynamics of gametic molecules in externally fertilizing marine organisms (see [83] for a recent review). Studies on the abalone sperm molecule *lysin* and its egg receptor *VERL* demonstrate the fact that male and female gametic proteins coevolve species-specific structures and affinities to maintain species-specific interactions and avoid cross-fertilization [83, 84, 88–90]. The sperm molecule *bindin* and its egg receptor are another classic example from sea urchins [83, 84, 88, 91]. While the evolutionary dynamics of the egg receptor for *bindin* remains obscure, the sperm protein *bindin* evolves rapidly and divergently in some genera but not in others [83, 84]. However, *bindin* does appear to have some involvement in reproductive isolation since its divergence correlates with the degree of gametic incompatibility between but not with time since species divergence [92]. In all likelihood, other molecules must be involved and there is a need for further characterization of such gametic and other sex and reproduction-related molecules in broadcast spawners. Once such molecules are identified, it will be interesting to correlate the evolution of egg-sperm interacting molecules to the patterns of hybrid incompatibility in these organisms. External fertilizing systems may provide a unique opportunity to assess the relative roles and genetic consequences of sexual selection and conflict in driving divergence of reproductive molecules and speciation. While research into reproductive molecules is at its inception in externally fertilizing systems, the sperm proteome of *Drosophila* has opened up exciting venues of research in reproductive biology [93–95]. As with other male-biased genes, sperm genes are underrepresented on the X chromosome and are nonrandomly clustered in the genome. While certain groups of sperm proteins, such as binding factors, do evolve rapidly, overall, the sperm proteome does not appear to be evolving fast, there is little evidence of positive selection, and there is widespread functional and structural constraint [94]. This is in stark contrast to seminal fluid proteins that evolve fast and are under selection. The contrasting evolutionary patterns of the two groups of male ejaculate proteins are interesting and are indicative of the complexity of reproductive processes, where crucial sperm-egg interacting proteins are sheltered but seminal fluids that accompany them interact with the general environment of the female reproductive tract and proteins therein are under strong selection. Future research on sperm-egg interacting proteins promises to increase our knowledge about the functional evolution of the male and female fertilization machinery and, more broadly, the evolutionary origins of sexual reproduction. 

### 4.3. SRR Genes and the Evolution of Hybrid Sterility

Haldane's rule (of speciation) points to the preferential appearance of hybrid sterility and and/or inviability in the heterogametic sex [82]. In flies and mammals, it is the males who are affected while in moths and birds it is the females [39, 96, 97]. The genetics of hybrid sterility and inviability have been of intense focus in speciation studies and a great deal of effort has been made in mapping and characterizing genes involved in hybrid “breakdown” [38, 39, 50, 98–106]. The evolution of hybrid sterility/inviability is explained by the Bateson-Dobzhansky-Muller incompatibility model which states that incompatibility is the result of independent evolution of genes in isolated populations [7, 106]. Haerty and Singh [27] showed that the genes showing breakdown in the hybrids are preferentially sex-related genes and that these genes evolve faster both in sequence and gene expression [27, 28, 107]. In the light of this it is interesting to note that all but a handful of the so-called “speciation genes” and hybrid-sterility genes are characterized by high sequence divergence; they are often sex-related genes or somatic genes that affect the sexual system [7, 38, 40, 41, 108]. 

The effects of genes are prone to change in response to incorporation of new mutants and during the course of speciation earlier mutations would have fewer interactions than older mutations (cascade effect). Thus it's entirely likely that the large effect “speciation genes” may have started as small-effect minor genes and have become elaborate in their genic interactions later through species-specific adaptation and evolution. In this scenario there is no conflict between the role of minor and major genes. Thus the effect of cascade evolution is not only that speciation would occur rapidly and precipitously but also that speciation genes would evolve in their average effect from being minor to major genes. So while in reality speciation may occur in an incremental manner through a combination of many minor genes, in postspeciation genetic investigations genes would often appear as major genes. This is an interesting scenario and we must find a way to approach this problem experimentally. 

### 4.4. SRR Genes and the Evolution of Development

 SRR genes provide new opportunities to mount comparative studies of the role of selection versus developmental constraints in evolution. For example, SRR genes have provided an excellent example of testing alternative explanations for Von Baer's third law. Von Baer observed that earlier stages of ontogeny were more conserved than later stages [109]. This was later interpreted to be the result of selection against changes in earlier stages of development, which could have cascading, deleterious developmental repercussions. Darwin on the other hand explained the conservation of morphology in earlier stages as being due to lack of opportunity for natural selection to act. Since natural selection results from changes in the environment, it follows that earlier, sessile stages that have not fully developed will have little opportunity to experience variation in the environment. Darwin further pointed out that secondary/sex-specific sexual traits appear when they are needed and this can be seen in the secondary sexual traits in animals. Two recent studies [28, 107] explored the relationship between expression level over ontogeny and rates of divergence and found support for both selection against deleterious cascading effects and Darwin's hypothesis: genes expressed during early stages show reduced divergence. However, the more rapid divergence of later, adult stages, is dominated by genes expressed in adult males, which are, as noted above, presumably diverging under the effect of directional (sexual) selection. As a result of these observations, Artieri et al. [107] proposed a model of divergence involving two factors playing dominant roles during different periods of development: conservation early and opportunity late. More of such studies should shed light on the relationship between evolution and development [110–113] as well as on the broader aspects of speciation and macroevolution beyond reproductive isolation. 

## 5. Sexual Conflict, Sexual Arms Race, and Sexual Selection

 While SRR research has provided a useful, complementary approach to study speciation, there is a need to explain the evolutionary forces driving pervasive rapid evolution of male and female reproductive genes. Initially rapid SRR gene evolution invoked the role of sexual selection by female choice but recent developments on Parker's [114] original theses revived the role of sexual conflict in explaining evolutionary changes in sexual systems [115]. Sexual dimorphism in higher organisms leads to sex-specific life styles, reproductive behaviours, and investments in sexual interactions. It is expected that these differences will lead to conflicts of interest between males and females and this conflict may work as a stimulus for “retaliatory” evolutionary changes known as “sexual arms races” [115–117]. A sexual arms race would require action and reaction on the part of both partners and thus it provides a test of the role of female choice, male-driven sexual selection, and of sexual arms race theories. Increasing empirical evidence suggests that sexual conflict may be pervasive [90, 115, 118–123] but much remains to be done particularly at the level of genes to substantiate how sexual conflict and sexual selection affect male and female genetic systems differently. Genomics provides the means to explore the molecular consequences of sexual arms races and associated sexual selection theories. Intersexual interactions, be they mutualistic or antagonistic, have the potential to drive population divergence in a self-accelerating manner and this may be one of the reasons why origins of diversity and speciation are much higher in higher organisms. Evo-devo studies will also help to answer the perennial question: during evolution and speciation what comes first—reproductive isolation or adaptive radiation? 

## 6. Conclusion

Since its inception 25 years ago, SRR gene research has rapidly evolved into a large coherent field in evolutionary biology, particularly influencing reproductive biology and speciation. The focus on SRR system studies has provided valuable mechanistic frameworks that directly relate to theories of how speciation occurs. It has emphasized the role of sexual selection in evolution, propelling research on how sexual selection and sexual conflict work at the population level. The genomics era has revolutionized SRR gene research and resulted in the characterization of rates of evolution and patterns of gene expression in reproductive transcriptomes. Rapid evolution is now commonly associated with reproductive genes but, in the future, work will be needed to understand the functions of rapidly evolving SRR genes and details of why they evolve rapidly and how their rapid evolution affects the rest of organismal biology. It will call for an integrated approach, unifying disparate fields of science, particularly biochemistry, genetics, ecology, and molecular biology. A key issue will be to explore the relationship between changes in gene sequence, gene expression, protein syntheses, and protein function in reproductive systems. Fundamental behavioural and ecological studies will be essential in explaining the nature of molecular changes associated with the reproductive systems. Selection in relation to sex, encompassing sexual selection in the strict sense, and in the broad sense is a large and growing area of research in evolutionary biology. Investigating the molecular consequences of sexual interaction and their role in speciation stands to open one of the most important areas of research bearing on the biology of sexual reproduction. 
